# Early cellular plasticity promotes progression and dissemination in pancreatic adenocarcinoma

**DOI:** 10.1007/s10555-026-10326-1

**Published:** 2026-03-12

**Authors:** Giulio Innamorati, Giorgio Malpeli, Luca Giacomello, Roberto Salvia, Thomas M. Wilkie

**Affiliations:** 1 Department of Surgical Sciences, Dentistry, Gynecology and Pediatrics, University of Verona, c/o GB Rossi General Hospital, P.Le L.A. Scuro, 37134 Verona, Italy; 2 Department of Life Science, Health, and Health Professions, Link Campus University, Rome, Italy; 3 Department of Engineering for Innovation Medicine, University of Verona, Verona, Italy; 4 Pharmacology Department, UT Southwestern Medical Center, Dallas, TX USA

**Keywords:** Pancreatic ductal adenocarcinoma, Cellular plasticity, Early cancer dissemination

## Abstract

Pancreatic ductal adenocarcinoma (PDAC) remains one of the most lethal malignancies, with limited therapeutic success and a persistently low 5-year survival rate. Despite significant advances in genomics and tumor biology, a fundamental challenge persists: to identify the elusive transformation from common benign pancreatic lesions to occasional malignant cellular identity. This review addresses a critical missing link in PDAC pathogenesis, focusing on when and where the switch to malignancy occurs, and why surgical intervention is often insufficient. We explore the biological and spatial–temporal evolution of precancerous lesions, such as PanINs and IPMNs, and examine how phenotypic plasticity and overlapping cellular programs—including squamous transdifferentiation, epithelial-to-mesenchymal transition (EMT), and acquisition of mesenchymal features—contribute to early dissemination, treatment resistance, and surgical failure. Recognizing and characterizing these early molecular events is essential for rethinking therapeutic strategies, identifying actionable biomarkers, and redefining the temporal window when curative intervention is feasible.

## Introduction

In countertendency to most cancers, the global prevalence of pancreatic ductal adenocarcinoma (PDAC) is rising. This trend is expected to persist, driven in part by demographic changes such as longer life expectancy and the growing prevalence of obesity and diabetes mellitus [4]. Despite advances in cancer biology and therapeutics, progress in understanding PDAC initiation and improving patient outcomes remains limited. Early-stage PDAC is almost always clinically silent and most patients present with systemic disease involving lymph nodes, peritoneum, liver, or lungs. Even when tumors appear localized, pancreatectomy with negative margins rarely prevents recurrence [5].

These clinical patterns indicate that dissemination frequently occurs before diagnosis. Micrometastatic cells, invisible to current imaging modalities, evade both surgical removal and standard chemotherapeutic regimens. This raises fundamental questions about the earliest biological events that enable KRAS‑mutant epithelial cells—shaped by inflammatory cues, stromal interactions, and desmoplastic remodeling—to acquire plasticity enabling neoplastic transformation and early escape. Which molecular programs drive the progression of low‑grade precursor lesions into invasive disease, and which cellular populations within the tumor or its microenvironment are responsible for this silent spread? Defining the identity and timing of the cells that disseminate from very early pancreatic lesions—long before a detectable primary tumor forms—is critical for developing effective interventions.

Emerging spatial and high-throughput multiomic technologies have reframed PDAC as a dynamic, spatially organized ecosystem rather than a linear, cell-autonomous process. Clonal evolution with progressive accumulation of driver mutations alone cannot account for the transition from preinvasive lesions to widespread metastasis. Instead, integrated experimental approaches highlight the roles of epigenetic regulation, microenvironmental selective pressures, and epithelial–mesenchymal plasticity. Transient, reversible shifts between sedentary and motile stress-resistant states allow small subpopulations of cells to disseminate and persist as dormant or slowly cycling tumor cells. Spatial transcriptomic and proteomic analyses further reveal that invasive fronts and metastatic niches harbor distinct stromal and immune architectures, reflecting a branched evolutionary trajectory shaped by a continuum of adaptive cell states [6, 7].

In this review, we summarize recent advances and outstanding questions regarding the genetic and molecular mechanisms that drive early tumorigenesis, malignant transformation, and metastasis in pancreatic cancer, with particular emphasis on the role of phenotypic plasticity in tumor invasion and dissemination. Emerging data offer the potential to identify dispersed tumor cells that evade detection by conventional morphological or bulk analytical methods, ultimately opening the door to earlier prognosis and more effective therapeutic strategies.

## Known drivers, obscure origins: the search for the metastatic progenitor in its provocative environment

**Fig. 1:**
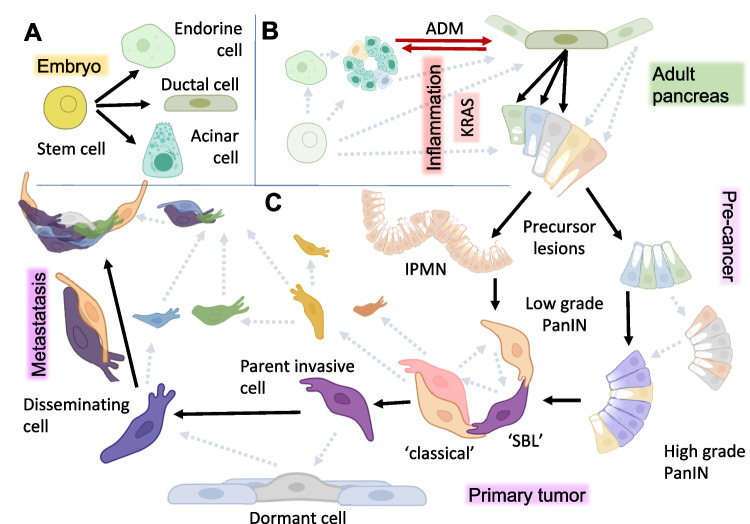
PDAC origin—Black arrows represent accepted steps while grey dashed arrows describe less supported steps implying metachronous parallel evolution. Varying colors represent heterogeneity present at all levels. **A** During embryonic development, stem cells give rise to ducts, islets, and acini (green arrows). **B** In adult life, uncharacterized adult stem cells may restore differentiated cells. Acinar cells show heterogeneity and the highest degree of plasticity [2]. Inflammatory insults can induce a reversible transformation of acinar cells into ductal-like cells (ADM, red arrows). Persistent growth factor and CREB signaling or KRAS lock the duct-like cells in their transdifferentiated state promoting PanIN formation. Heterogeneity, exemplified by different colors, already present at the single-cell level in precancer lesions cannot be simply described by a phylogenetic tree ([3] and main text). Directly or indirectly, precancerous cells in PanIN or IPMN could originate from any pancreas cell lineages (dashed arrows). **C** The inactivation of other oncosuppressors further promotes progression. Among multiple independent precancerous lesions that accumulate in the aging pancreas, malignancy arises only rarely originating the primary tumor. When it does, early cancer development is characterized by heterogeneous cellular phenotypes and genotypes, without a clearly defined branching phylogenetic pattern. Sometime along this entire process, before clinical symptoms appear, unidentified cancer cells can disseminate beyond the pancreas, spreading as elusive and heterogeneous populations in lymph nodes, the liver, the lungs, the peritoneal cavity, and the bone marrow. Dormant cells within micrometastases may serve as a reservoir for cancer recurrence without ever becoming clinically apparent. The evolutionary process sees multiple subclones emerge early, compete for fitness, and differentially seed local invasion and distant metastases rather than following a single linear trajectory. Eventually, classical and SBL molecular phenotypes are preserved at the metastatic site

Gain-of-function mutations in KRAS and subsequent loss-of-function mutations in tumor suppressor genes drive early tumorigenesis and subsequent progression in over 90% of PDAC [8]. However, recent advances in spatial biology and high-throughput omics technologies (reviewed in [3]) have challenged the traditional view that PDAC arises from a single clonal origin. Although pluripotent cancer stem cells are widely believed to drive disease dissemination, in PDAC their precise origins remain obscure, and no consensus has been reached regarding specific markers [9]. During embryonic development, three main lineages derive from pluripotent stem cells: acinar, ductal, and multiple endocrine subtypes (Fig. 1A). Pancreatic adult stem cells have long been suspected as progenitors for the three lineages as well as neoplastic cells; however, their identity, localization, and defining markers remain controversial [10]. An alternative hypothesis posits differentiated cells acquiring abnormal plasticity and reverting to a progenitor-like state [2, 11].

**Fig. 2:**
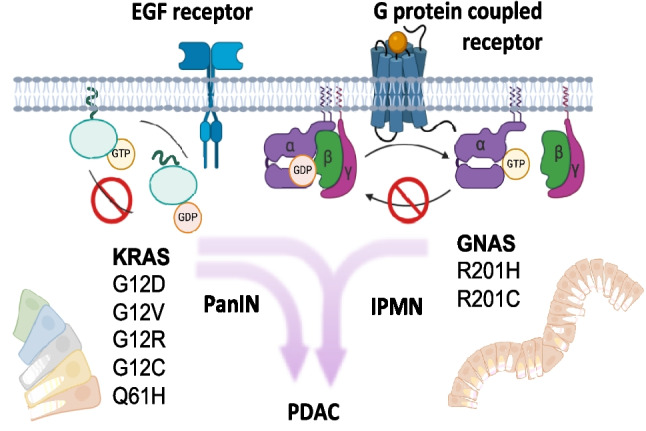
Oncogenic G-proteins driving PDAC. Highly specific hotspot mutations of G-proteins slow down GTPase activity, preserving and enhancing receptor downstream oncogenic signaling. Only a limited number of amino acid substitutions confer gain rather than loss of function. In pancreatic precancer lesions, GNAS mutations are specific for IPMN, often combined with KRAS, which is a driver in both PanIN and IPMN [1]

KRAS mutations (Fig. 2) and environmental insults can induce acinar-to-ductal metaplasia (ADM), a process in which acinar cells transdifferentiate into ductal-like cells. ADM is facilitated by inflammation and is reversible upon elimination of the inciting insult. The precise downstream mechanisms that drive the development of irreversible benign lesions remain undefined, including whether these lesions can arise from all pancreatic cell lineages (Fig. 1B) [2, 10, 12]. The prevalence of the most common pre-cancer formation, pancreatic intraepithelial neoplasm (PanIN), in the general adult population is estimated to be 60% to 86%, gradually accumulating in individuals over age 30 [13, 14]. Most precancerous lesions are driven by any one of several KRAS hotspot mutations that inhibit GTP hydrolysis, thus causing persistent activation. Analogous hotspot mutations in GNAS (Fig. 2) tend to develop intraductal papillary mucinous neoplasms (IPMN) which are larger, less aggressive cysts [15–17]. Three-dimensional spatial analysis revealed that grossly normal tissue can harbor hundreds of multifocal PanIN [18]. IPMNs are less common, with prevalence increasing with age and reaching about 10% in the general population over 50. A significant increase in PDAC development was observed only in patients with cysts classified as high risk according to Fukuoka criteria. Cysts without high-risk stigmata or worrisome features were associated with PDAC risk comparable to that of patients without cysts [19].

Both GNAS and KRAS driver mutations, due to the specificity of their base substitutions, are readily detectable by DNA sequencing in cancer tissue [20]. However, intratumoral heterogeneity in PDAC reflects diverse combinations of KRAS [21] and GNAS [22] mutations, implying that multiple precancerous lesions can arise independently and progress in parallel toward high-grade dysplasia. Morphological reconstruction has even revealed distinct KRAS variants within single PanINs, consistent with a multifocal intra-lesional origin rather than expansion from a single dominant clone [18]. One mechanism proposed to account for the metachronous emergence of such mutations is regional depletion of deoxynucleotide triphosphate (dNTPs) pools – for example, secondary to metabolic stress [23] – which promotes error-prone repair and genomic-wide mutagenesis, including oncogenes such as KRAS [24]. Crucially, these genetic events arise within a dynamic stromal and metabolic microenvironment that modulate cell state: nutrient limitation, extracellular matrix remodeling, and stromal signaling can foster phenotypic plasticity, enabling disseminating cells to adopt invasive or survival programs despite limited clonal expansion. Morphologic analysis suggests early lesions arise as interspersed neoplastic cells within apparently normal ductal cells and surrounding fibroblasts. As these lesions grow and branch, they displace acinar cells, entrap islets, and encircle peripheral nerves. Concurrent pancreatitis contributes an inflammatory cicatricial matrix, typical of the tumor microenvironment and variably populated by fibroblasts, lymphocytic, and myeloid infiltrate [25]. Cancer cells are marked by oncogenic mutations but not the stromal cells of the tumor microenvironment. If GNAS and KRAS mutations represent early events, then genetic heterogeneity emerges during the initial phases of transformation, and PDAC appears to evolve over a diffused network of multiclonal and convoluted PanINs. Together, these observations link multifocal mutagenesis and microenvironmental pressures to the emergence of phenotypically plastic cells capable of early dissemination. The overall scenario sees the tumor mass generated by a mosaic of precancer cells with distinct signaling properties. Subsequent mutations in tumor suppressors (e.g. *TP53, SMAD4, CDKN2A*) and copy number variation (CNV) alterations drive toward malignant progression.

## Marked for departure: spotting the pre-metastatic pioneers

Studies using single-nucleotide variants and insertions/deletions to analyze microdissected PanINs and distant PDAC lesions within the same pancreas [26] have revealed:

- Distinct progeny suggesting synchronous growth.
- Close proximity when PanINs share all driver mutations.
- Common ancestors indicating branching during progression.

These findings suggest that multiple precancerous cells, morphologically appearing as PanIN and already carrying the most common driver mutations, spread and progress through ducts [26]. This raises concerns about the specificity of early diagnostic tests based solely on KRAS or GNAS mutations, as these alterations are also found in benign precursor lesions. In fact, mutant DNA has been detected in the plasma of healthy individuals and patients with pancreatitis [27]. In addition to these potential confounding effects, a blood-based diagnostic biomarker that detects PDAC at a surgically curable stage must identify cancer when lesions are still subcentimeter. At this stage, the mass will contain relatively few tumor cells, show minimal necrosis, and thus release a very small amount of cell free DNA only modestly higher than in advanced precursor lesions. Even with current high-performance circulating tumor DNA assays, at such an early stage, the probability of detecting tumor-derived circulating DNA in peripheral blood is very low because mutant fragments are highly diluted within the total cell-free DNA pool. Detection of actively secreted proteins or small vesicles are expected to be more sensitive reporters. At present, biomarkers such as Ca19.9 are only informative for monitoring disease progression once a significant tumor burden is established within the pancreas and metastatic sites [28]. Although it is widely accepted that cancer cells can enter lymphatics vessels or the bloodstream, the precise identity and mode of transit of these cells remain poorly defined: it is unclear whether they disseminate as single cells or as multicellular clusters; it has even been proposed that dissemination may involve fusion between tumor cells and macrophages [29]. Consequently, research should prioritize detailed characterization of early intraductal dissemination and the development of highly sensitive biomarkers capable of detecting subcentimeter, surgically curable lesions.

## The struggle of tracing ancestors of disseminated cells

Characterizing asymptomatic progression to systemic disease represents a major challenge for researchers since authentic early-stage human samples—particularly from the pancreas—are virtually inaccessible. The collection of human tissue samples either comes from advanced cases or from autoptic material that can only provide incidental pre-neoplastic lesions that only in rare and unpredictable cases may have evolved to cancer during subsequent years. As a result, most research has relied on tissue specimens derived from samples of clinically manifest illness collected from patients who underwent surgical procedures, leaving the timing and cellular origin of malignancy substantially unresolved. Resected specimens inevitably fail to accurately represent the early-stage disease from which disseminating cells originated.

Two methodological approaches may overcome this issue. One is a periodic collection and storage of samples and clinical information from a large population of ‘healthy individuals’. Rare cases developing the disease will have available a historical set of clinical data and samples. A similar massive effort to cover the history of PDAC insurgency from the origin is being undertaken by the PRECEDE consortium (www.precedestudy.org) focused on high-risk individuals limited to relatively small plasma aliquots, or other specimens that can be collected by non-invasive procedures, lacking early tissue samples.

Otherwise, much like archaeologists, researchers must reconstruct the phylogenesis of the disease, delineating—at the single-cell level—the processes of de-differentiation and re-differentiation that drive malignant progression. CNV analyses of matched primary tumors and metastases across multiple organs in the same patient have failed to reveal a unified evolutionary trajectory. Instead, multiple phylogenetic structures have been inferred, indicating complex and branched patterns (Fig. 1C) of cancer cell dissemination [30].

Recent advances, particularly in single-cell analysis and spatial biology, have revealed that PDAC is a highly heterogeneous and multifocal disease within individual patients [31–33]. Perhaps for this reason the efforts to personalize therapy based on mutational profiles have not yielded the anticipated clinical benefit [34]. The fateful step and timing of when disease disseminates remain to be clarified.

## Transformed cells leave home: the escape from primary tumors

If in the context of the acquisition of cellular plasticity, as a seminal event for cancerogenesis, genomic analysis has failed to yield a comprehensive model of PDAC onset and progression. Recent advances have emerged from efforts to characterize the intricate crosstalk between gene transcription and the extracellular microenvironment. Among these, EMT and inflammation are recognized as early and interrelated events in tumorigenesis [35]. Microenvironment-induced EMT promotes epithelial cell migration and contributes to cellular transformation and invasiveness, thereby playing a pivotal role in the initial steps of dissemination. In the earliest stages of pancreatic neoplasia, PanIN lesions arise within a stroma enriched in nerve fibers and collagen deposition, which progressively evolves into the dense desmoplastic matrix that defines PDAC histopathology (reviewed in [36]). Importantly, the structural properties of collagen I differ depending on its source: fibroblasts mainly synthesize the heterotrimeric isoform, whereas transformed epithelial cells produce the homotrimeric variant. These differences critically modulate immune cell recruitment and enhance invasive behavior [37].

Similar to human disease, in most genetically engineered mouse models (GEMMs), PDAC initiation depends on orthotopic expression of KRAS^G12D^. Acinar cells expressing KRas isolated from murine pancreas and embedded in collagen initiate ADM under low-stiffness conditions. Yet, they reprogram transcription to express ductal/progenitor markers only upon increasing rigidity after YAP/TAZ nuclear translocation [38]. Tumor progression accelerates when KRAS^G12D^ expression is combined with loss of tumor suppressors such as Tp53 (KPC model) and/or *Ink4a* (KIC model). Although these models artificially shorten early disease stages and may not fully capture the genetic heterogeneity of human PDAC—particularly that of disseminated tumor cells [39]—they demonstrate that inhibiting collagen-fibers deposition can prevent neoplastic lesion formation [38].

Mechanistic studies in GEMMs further indicated that EMT and cell dissemination occur surprisingly early, before the appearance of overt carcinoma. KRAS-positive PanIN or ADM cells have been observed to delaminate and enter the bloodstream, consistent with early systemic spread [40]. More than a decade ago, lineage-tracing experiments in KPC mice revealed that pancreatic-derived mesenchymal-like cells colonize distant organs such as the liver long before histological detection of malignancy. Blocking inflammation in these models prevented PanIN formation, suppressed EMT, and abolished early cell dissemination [35]. A more recent study introduced a TFF1 knockout in the KPC background to enhance EMT features [41]. Tagged cells disseminated more efficiently at advanced PanIN or early PDAC state than later stages, supporting the hypothesis formulated for other malignancies, particularly for ductal carcinomas (breast, melanoma, esophageal, colorectal, ovarian), that neoplastic cells can spread early and remain dormant and undetected at distant sites for several years before clinical manifestation [42]. Remarkably, disseminated cells exhibited phenotypic plasticity and even expressed tissue-specific markers, resembling hepatocytes in the liver, pulmonary epithelium in the lung, and leukocytes in the bone marrow [41]. Complementary GEMM, designed to distinguish EMT-proficient epithelial cells from those acquiring a mesenchymal phenotype, confirmed that mesenchymal plasticity occurs in PanIN. In this model, the activation of EMT program results in genomic instability, chromothripsis, and the acquisition of metastatic competency [43]. Thus, PDAC dissemination is likely to occur long before the formation of a solid mass.

Clinically, parallel evidence supports early systemic spread in patients. While bone metastases are uncommon, extensive studies demonstrated early dissemination of neoplastic cells in the bone marrow, often carrying a reduced burden of driver mutations [42]. *KRT7/8*-positive cells have been detected in roughly one-third of cases and correlate with poorer prognosis and smaller yet more aggressive primary tumors [44]. These findings suggest that dormant micrometastases, seeded during the earliest phases of tumorigenesis, may persist in quiescent niches. Though clinically silent, such lesions can shed cells that ultimately undermine the efficacy of surgical resection of tumors deemed localized [45].

## The migratory phenotype: hallmarks of cellular journey

**Fig. 3:**
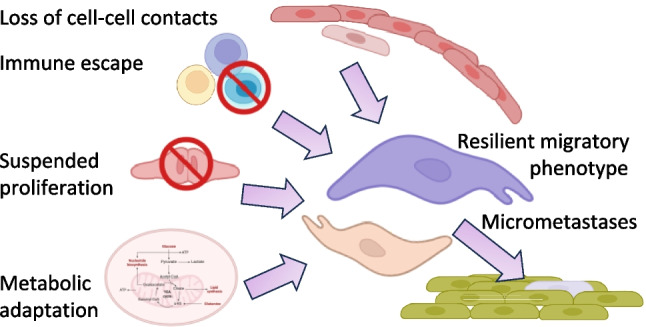
EMT and dissemination. Epithelial cells undergo loss of tight junctions and adopt migratory stress-resistant phenotypes that permit dissemination and subsequent engraftment at distant sites, where new contacts are formed. In PDAC, occult micrometastases maintain disease dormancy. Later reactivation produces liver and peritoneal metastases, which are the principal drivers of morbidity and mortality

Depending on microenvironmental cue and cell-state transition, PDAC cells can employ different migration strategies including collective, mesenchymal, and amoeboid migration [46]. EMT endows cells with the ability to migrate, survive under stress, and evade immune surveillance, and may not form a manifest tumor mass. EMT adaptation involves several key and interdependent changes for dissemination (Fig. 3):

- **Loss of cell–cell adhesion** and reorganization of junctional complexes, cytoskeletal remodeling, and microenvironmental interactions are required to migrate from the primary tumor site. Moving through dense ECM is highly energy demanding, with rapid ATP turnover at actomyosin structures and focal adhesions. In addition, cells can either migrate individually or collectively following leader cells. Higher energy costs paid by the leader cells are compensated by transient upregulation of glycolysis or even genetic modifications to upregulate oxidative phosphorylation [47].
- **Metabolic reprogramming** to support movement and survival under adequate oxygen conditions or nutrient- and oxygen-deprived environments. Reframing the concept of the Warburg effect, PDAC cells can exploit glycolysis-high and OXPHOS-high states to reconfigure glucose, glutamine, lipid, and redox pathways in response to mechanical, nutrient stresses or drug exposure [48]. As a result, lactate resulting from glycolysis can trigger signaling further supporting survival, invasion, metastasis, angiogenesis and immune escape [49]
- **Alteration of surface epitopes** and immune escape by multiple mechanisms reviewed in [50] includes acidosis derived from glycolysis which inhibits effector cells such as CD8 + T and NK cells, while supporting immunosuppressive regulatory T cells and myeloid-derived suppressor cells [51].

- **Modulation of proliferation**, with reduced division during migration (Fig. 3).

In PDAC, these features are characteristic of the squamous/basal-like (SBL), the molecular subtype identified in the attempt to stratify patients at the transcriptomic level [52].

## Diverging destinies: the “classical” and “squamous” progeny

Adenocarcinoma or squamous cell carcinoma are the two major histological subtypes of epithelial carcinoma. However, adenocarcinoma can acquire squamous properties. In PDAC, pathologists have long noted a squamous cell component in a subset of tumors. When this component exceeds one-third of the tumor mass, the diagnosis is revised to adenosquamous carcinoma, a variant associated with increased aggressiveness. Proteomic comparison across distinct tumor types has identified transversal markers that distinguish squamous carcinoma from adenocarcinomas cells irrespective of tissue origin [53]. This suggests a shared transcriptional reprogramming that drives the transition from an epithelial to a squamous phenotype.

Unsupervised transcriptomic analyses aimed at finding prognostic molecular signatures produced multiple subclassifications of gastrointestinal malignancies. Across these efforts, a mesenchymal molecular pattern consistently overlapped with poorer prognosis relative to epithelial subtypes [54].

In PDAC, molecular taxonomy evolved from early three‑subtype schemes in the 2010 s to more refined multi‑subtype classification with advances in genomics, transcriptomics, and single‑cell technologies. These definitions have eventually converged into two major transcriptomic subtypes: the classical and the SBL (reviewed in [55] which also explains how additional subtypes may represent contaminations from other lineages or intermediate stages). KPC mouse models indicate that mutations in acinar cells preferentially give rise to the classical subtype, whereas SBL subtype cells would arise from ductal cells [56]. However, single-cell analyses have shown that both subtypes coexist within the same tumor, contributing to intratumoral heterogeneity, with the SBL component associated with poorer prognosis and greater metastatic potential [3, 57].

Microdissection of morphologically distinct lesions (200–500 cells) by Di Chiaro et al. identified three "morpho-biotypes": gland-forming, non-gland-forming, and poorly differentiated variants [28]. RNA analysis linked these morphologies to molecular subtypes: gland-forming lesions were predominantly classical-like, while poorly differentiated and signet-ring cell–containing lesions were enriched for SBL features [58].

The SBL subtype exhibits several traits associated with aggressive disease, including:

- Immune evasion;
- Adaptation to hypoxia, even under normoxic conditions;
- Enhanced metastatic potential;
- Therapy resistance;
- Worse clinical outcome [59].

Although EMT has been reported to be activated in both classical and SBL subtypes, the underlying transcriptional programs differ [59]. The squamous transcriptional pattern is characterized by abundant extracellular matrix synthesis and EMT, consistent with a migration-prone phenotype. The GEMM study referenced above, designed to dissect EMT dynamics, indicates that EMT facilitates the acquisition of mesenchymal traits along with progressive genomic instability, resulting in genomic aberrations and elevated transcriptional entropy; conversely, EMT inhibition reduces phenotypic heterogeneity and preserves ductal-like morphology and classical-like transcriptional profile. Thus, EMT contributes to generating multiple transcriptomic trajectories that enhance tumor heterogeneity [43]. It is therefore plausible that the SBL phenotype reflects an EMT-driven evolution of a high-fitness population with increased metastatic potential and therapy resilience.

Despite this, transcriptional profiling by Park et al. [59] and Pei et al. [30] reveals both classical and SBL components persist in metastatic lesions, with the classical subtype cells showing only a relative preference for lung colonization over liver metastasis [30]. These findings suggest that expression subtype acquisition is not tied to the clonal architecture inferred from genomic analysis and that the relative capacity of each subtype to disseminate remains incompletely understood. Aiello et al. associated the classical phenotype with a partial EMT program and SBL with an alternative, more complete EMT program that—through transcriptional regulation—drives single-cell migration of SBL cells [60]. The same lineage-labeled KPC model indicates that the classical subtype loses the epithelial characteristics by internalizing membrane proteins such as E-cadherins. By retaining intercellular cohesion, tumor spheres derived from classical cells were reported to invade a 3D matrix via collective cell migration, a process recently shown to be facilitated by aligned collagen, which reduces the energetic demands for migration in PDAC BxPC-3 cell line [61].

An additional layer of complexity arises from the need for disseminated cells to adapt to the local microenvironment, which can obscure their origin. Spatial adaptation of PDAC cells has been reviewed in detail by Maddipati, including implications for therapy [62]. Even at sites where metastases are not commonly detected, engrafted cells can acquire organ‑specific genes, as mentioned above for most common metastatic sites (liver and lungs) [63]. For example, PDAC cells infiltrating the duodenal wall have been shown to respond to local cues, integrating into the native epithelium and adopting classical phenotypic features while preserving the mucosal architecture [64]. Reproducing the true complexity of pancreatic cancer cell spread in experimental models is evidently a formidable challenge.

## Cultivating complexity—why organoids fall short as patient stand-ins

While the transition between classical and squamous lineages defines a clinical trajectory of the disease, our ability to model this plasticity remains limited; even our most advanced 3D organoids often fail to capture the TME-driven signals that trigger these lineage shifts in the patient. The relevance of PDAC experimental models (including combinations of 3D, slice cultures, tumor-on-a-chip, and animal models) to orient precision medicine strategies was recently reviewed in ref. [65]. Patient-derived xenografts obtained from tumor fragments (PDX) or tumoroids (PDO) are widely used and currently the best models for personalized therapies. However, inevitably, murine avatars are established using cells harvested from the primary tumor post-diagnosis, at a point when the malignancy has already metastasized beyond the tissue of origin. Consequently, their fidelity in recapitulating the tumorigenic inception or in forecasting systemic eradication of neoplastic cells remains unvalidated. Furthermore, xenografted human cells disseminate within the murine host in the absence of critical microenvironmental elements, including the dysplastic extracellular matrix, stromal fibroblast populations, and the patient's autologous immune response. The inevitable loss of the original microenvironment in *ex vivo* models dramatically affects heterogeneity.

From a clinical implementation standpoint, turnaround time remains a major bottleneck: the establishment, expansion, and pharmacotyping of patient‑derived PDAC organoids often exceed the clinical window available for first‑line treatment decisions. Nevertheless, the field is rapidly advancing its ability to preserve tumor complexity [66] and is expected to ultimately inform and improve therapeutic strategies.

Single-cell analysis of tumors derived from a modified version of the KPC mouse model suggested a continuum going from the classical transcriptional subtype, enriched in mucins, towards mesenchymal transitioning through a basal-like intermediate state enriched in keratins and laminin. Two-dimensional cell culturing skews towards the BSL phenotype as opposed to 3D organoids culture. However, plasticity was conserved, as in both models heterogeneity could be restored by orthotopically implanting cells in NSG mice [67]. Organoids studies have provided further insights into subtype plasticity. Early-stage organoids display cystic morphology, Wnt-dependence, and classical transcriptomic features whereas advanced organoids exhibit Wnt-independence and express squamous markers such as TP63, KRT5, and KRT6A [68]. Standard organoid culture conditions include Wnt3a which promotes the canonical signaling pathway modulating βcatenin regulated transcription. In contrast, non-canonical Wnt ligands, such as Wnt7a, promote the non-canonical pathway mediated by planar cell polarity and Ca^2+^ signaling upstream transcriptional programs associated with motility and metastasis. Autocrine production and the inner core of a filled organoid could recreate the conditions for SBL cells. This suggests that current protocols based on Wnt3a, R-spondin, epithelial differentiating agents like noggin, and normoxia may facilitate the classical phenotypes, favoring initial proliferation, gland-forming morphology, and underrepresenting the SBL subtype [69, 70]. Conversely, within the hypoxic inner core of a filled organoid, it is plausible to hypothesize that autocrine factor production, coupled with minimal exposure to exogenous bovine serum components, more accurately recapitulates the microenvironmental conditions conducive to the emergence of the SBL phenotype [71] and cell heterogeneity. Nonetheless, such culture conditions are unlikely to reproduce the full spectrum of molecular divergence in systemic disease from which the patient-derived organoid originated [72]. Improving culture models to manipulate transdifferentiation of the classical and SBL subtypes remains challenging, particularly due to the difficulty in replicating microenvironmental cues, including mechanical tension that has been implicated in triggering malignant transformation [73].

## Targeting the shifting target: how plasticity and subtyping could redefine therapy

**Fig. 4:**
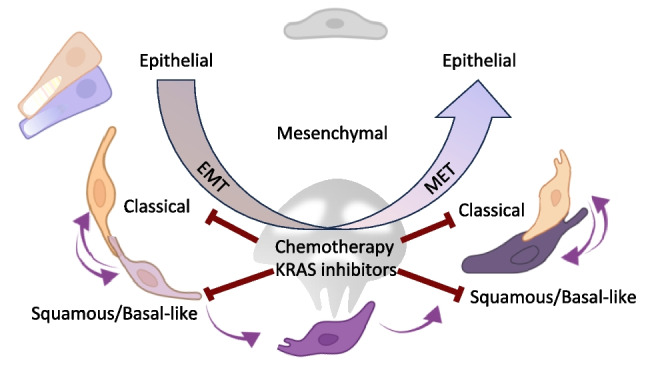
Vulnerability of a dynamic phenotype. Classical vs. BLS largely map onto epithelial vs. mesenchymal ends of the EMT–MET spectrum, but both signatures sit on a shared, plastic continuum enriched for hybrid (partial EMT) states rather than two discrete and non-overlapping programs. In practice, BLS signatures are strongly EMT-skewed, while classical signatures are epithelial/MET-skewed, with single cells co-expressing elements of both programs. An appropriate combination of therapies could reveal more effective targeting SBL and classical

While the precise relationship between classical and SBL subtypes remains unresolved, several scenarios are plausible. The SBL phenotype may arise through subclonal evolution from classical, glandular tumors [74]. Alternatively, the two subtypes may originate independently yet co-evolve and co-disseminate, potentially under the influence of SBL lineages; for instance, ΔNp63α—a marker and driver of squamous differentiation and the SBL phenotype—has been shown to promote EMT and collective migration in basal-like breast cancer [75]. Finally, the balance between classical and SBL dissemination may simply reflect plasticity driven by multiple factors, including passaging, oxygen tension and loss of cancer-stromal cell interactions within a microenvironment that changes during dissemination, dormancy outbreak or co-evolve in the primary site [30, 76] First-line systemic regimens are FOLFIRINOX (folinic acid, 5-fluorouracil, irinotecan, oxaliplatin) and gemcitabine plus nab-paclitaxel with a similar overall survival benefit between the two regimens. According to the COMPASS study, locally advanced or metastatic classical tumors responded better to modified FOLFIRINOX than basal-like, which may have a relatively better response to gemcitabine/nab-paclitaxel. However, only 20% of patients were “basal-like” and all at stage IV; the impact of differentiating treatment based on classical vs. SBL subtypes remains extremely limited [55, 77]. On the other hand, molecular profiling may become essential in positioning treatment with novel drugs that target KRAS and its downstream signaling. Great expectations are raising with the development of MRTX1133, which targets the G12D mutation predominant in PDAC, whereas G12C predominates in lung cancer and warrants the use of Sotorasib and Adagrasib. Unfortunately, a major limit to KRAS inhibition efficacy is the rapid emergence of drug resistance [78]. Secondary mutations in KRAS or other drivers are found involved in a limited number of cases. Given the relatively short temporal window of resistance development, these mutations likely exist prior to first round treatment, and cellular lineage plasticity adds to subclonal heterogeneity associated with compensatory reprogramming of signaling and metabolic pathways [79, 80]. Recent data from mouse and human PDAC samples revealed BSL cells are more sensitive to KRAS inhibition, compared to classical cancer cells, which appeared to become the reservoir for disease relapse [80, 81] (Fig. 4).

If BSL and classical are two faces of the same medal, treatment options should consider both within a timeframe that reduces the expansion of drug-tolerant cell populations. In this perspective, a practical classification of tumor subtypes in clinical settings becomes key. Feasibility barriers in PDAC molecular subtyping arise from the high cost, limited accessibility of comprehensive real-time genomic profiling, and viable biopsy availability. Immunohistochemistry is limited by inherent subjective variability, poor cancer cellularity, and only EUS will be available for neoadjuvant therapy. To circumvent these issues and develop a standardizable approach for implementing PDAC subtype classification, the Purity Independent Subtyping of Tumors (PurIST) algorithm was obtained by machine-learning–based, single-sample classifier. It evaluates relative expression ratios across eight gene pairs derived from tumor-intrinsic genes [46]. A test based on pancreatic tissue appears as feasible and clinically meaningful for advanced, treatment-naïve patients. Monitoring cell plasticity and the efficacy of treatment schemes designed to tackle the disease on multiple fronts would strongly benefit from noninvasive and repeatable tests.

## Conclusion

Extensive efforts to refine molecular signatures specific to malignant cells have led to the adaptation of concepts such as EMT, mesenchymal identity, and squamous differentiation in PDAC. These overlapping features reflect the remarkable cellular plasticity of PDAC, underlie the poor therapeutic response, and highlight the need for a clear understanding of tumor evolution. Recent advances in spatial biology and single-cell analysis suggest a paradigm shift is needed—one that prioritizes identification and treatment of asymptomatic disease and micrometastases.

Historically, research has focused on tissue samples and PDAC models that refer to advanced stages of disease within the pancreas. It remains difficult to obtain human tissue samples representative of the asymptomatic phase, between the preinvasive lesions and the authentically localized tumor. While GEMMs remain one of the most informative tools for studying early PDAC progression, their reliance on specific driver mutations fails to fully capture the genetic and phenotypic complexity of human disease. The limitations of GEMMs have been highlighted by the repeated failure of clinical trials based on preclinical findings from these models, particularly in recapitulating the full spectrum of metastatic behaviour [82].

The rapid evolution of three-dimensional models, such as organoids and tumoroids, offers the potential to incorporate patient-derived precancerous and stromal cells to better reflect the complexity of immune interactions and stromal dynamics. When integrated with spatial biology insights and advanced *in vivo* models, like GEMMs and PDXs, these systems provide a powerful framework for decoding cellular phenotypic fluidity.

This multimodal approach is essential for understanding asymptomatic dissemination and eradicating the micrometastases that currently limit the efficacy of surgical interventions.

## Data Availability

No datasets were generated or analysed during the current study.

## Abbreviations

PDAC: Pancreatic Ductal AdenoCarcinoma
EMT: Epithelial-to-mesenchymal transition
ADM: Acinar to ductal metaplasia
PanIN: PANcreatic Intraepithelial Neoplasm
IPMN: Intraductal papillary mucinous neoplasm
CNV: Copy number variation
GEMM: Genetically engineered mouse models
SBL: Squamous/basal-like
PDX: Patient-derived xenograft

## References

[1] Furukawa, T., Kuboki, Y., Tanji, E., Yoshida, S., Hatori, T., Yamamoto, M., Shibata, N., Shimizu, K., Kamatani, N., & Shiratori, K. (2011). Whole-exome sequencing uncovers frequent GNAS mutations in intraductal papillary mucinous neoplasms of the pancreas. *Scientific Reports,**1*, Article 161. 22355676 10.1038/srep00161 PMC3240977

[2] Zhang, X., Du, Y., Behrens, A., & Lan, L. (2025). Emerging insights into lineage plasticity in pancreatic cancer initiation, progression, and therapy resistance. *Developmental Cell,**60*(18), 2391–2406. 40987275 10.1016/j.devcel.2025.07.002

[3] Barthel, S., Falcomata, C., Rad, R., Theis, F. J., & Saur, D. (2023). Single-cell profiling to explore pancreatic cancer heterogeneity, plasticity and response to therapy. *Nature Cancer,**4*(4), 454–467. 36959420 10.1038/s43018-023-00526-x PMC7615362

[4] Klein, A. P. (2019). Pancreatic cancer: A growing burden. *The Lancet Gastroenterology & Hepatology,**4*(12), 895–896. 31648975 10.1016/S2468-1253(19)30323-1 PMC7376745

[5] Ingaldi, C., D’Ambra, V., Ricci, C., Alberici, L., Minghetti, M., Grego, D., Cavallaro, V., & Casadei, R. (2024). Clinicopathological predictive factors in long-term survivors who underwent surgery for pancreatic ductal adenocarcinoma: A single-center propensity score matched analysis. *World Journal of Surgery,**48*(12), 3001–3013. 39542862 10.1002/wjs.12397 PMC11619735

[6] Khaliq, A. M., Rajamohan, M., Saeed, O., Mansouri, K., Adil, A., Zhang, C., Turk, A., Carstens, J. L., House, M., Hayat, S., et al. (2024). Spatial transcriptomic analysis of primary and metastatic pancreatic cancers highlights tumor microenvironmental heterogeneity. *Nature Genetics,**56*(11), 2455–2465. 39294496 10.1038/s41588-024-01914-4

[7] Xu, Y., Wang, X., Li, Y., Mao, Y., Su, Y., Mao, Y., Yang, Y., Gao, W., Fu, C., Chen, W., et al. (2024). Multimodal single cell-resolved spatial proteomics reveal pancreatic tumor heterogeneity. *Nature Communications,**15*(1), Article 10100. 39572534 10.1038/s41467-024-54438-0 PMC11582669

[8] Kato, S., Lippman, S. M., Flaherty, K. T., & Kurzrock, R. (2016). The conundrum of genetic “drivers” in benign conditions. *Journal of the National Cancer Institute*. 10.1093/jnci/djw036 27059373 10.1093/jnci/djw036 PMC5017937

[9] Evan, T., Wang, V. M. Y., & Behrens, A. (2022). The roles of intratumour heterogeneity in the biology and treatment of pancreatic ductal adenocarcinoma. *Oncogene,**41*(42), 4686–4695. 36088504 10.1038/s41388-022-02448-x PMC9568427

[10] Zheng, C., Wang, J., Wang, J., Zhang, Q., & Liang, T. (2024). Cell of origin of pancreatic cancer: Novel findings and current understanding. *Pancreas,**53*(3), e288–e297. 38277420 10.1097/MPA.0000000000002301 PMC11882172

[11] Burclaff, J., & Mills, J. C. (2018). Plasticity of differentiated cells in wound repair and tumorigenesis, part I: Stomach and pancreas. *Disease Models & Mechanisms*. 10.1242/dmm.033373 10.1242/dmm.033373 PMC6078397 30037967

[12] Li, X., He, J., & Xie, K. (2022). Molecular signaling in pancreatic ductal metaplasia: Emerging biomarkers for detection and intervention of early pancreatic cancer. *Cellular Oncology (Dordrecht, Netherlands),**45*(2), 201–225. 35290607 10.1007/s13402-022-00664-x PMC12978117

[13] Matsuda, Y., Furukawa, T., Yachida, S., Nishimura, M., Seki, A., Nonaka, K., Aida, J., Takubo, K., Ishiwata, T., Kimura, W., et al. (2017). The prevalence and clinicopathological characteristics of high-grade pancreatic intraepithelial neoplasia: Autopsy study evaluating the entire pancreatic parenchyma. *Pancreas,**46*(5), 658–664. 28196020 10.1097/MPA.0000000000000786

[14] Longnecker, D. S., & Suriawinata, A. A. (2022). Incidence of pancreatic intraepithelial neoplasia in an autopsy series. *Pancreas,**51*(4), 305–309. 35775638 10.1097/MPA.0000000000002027

[15] Tan, M. C., Basturk, O., Brannon, A. R., Bhanot, U., Scott, S. N., Bouvier, N., LaFemina, J., Jarnagin, W. R., Berger, M. F., Klimstra, D., et al. (2015). GNAS and KRAS mutations define separate progression pathways in intraductal papillary mucinous neoplasm-associated carcinoma. *Journal of the American College of Surgeons,**220*(5), 845-854 e841. 25840541 10.1016/j.jamcollsurg.2014.11.029 PMC4409519

[16] Gaujoux, S., Parvanescu, A., Cesaretti, M., Silve, C., Bieche, I., Rebours, V., Levy, P., Sauvanet, A., & Cros, J. (2019). GNAS but not extended RAS mutations spectrum are associated with a better prognosis in intraductal pancreatic mucinous neoplasms. *Annals of Surgical Oncology,**26*(8), 2640–2650. 31025231 10.1245/s10434-019-07389-6

[17] Evans, J., Shivok, K., Chen, H. H., Gorgov, E., Bowne, W. B., Jain, A., Lavu, H., Yeo, C. J., & Nevler, A. (2025). Correlation of GNAS mutational status with oncologic outcomes in patients with resected intraductal papillary mucinous neoplasms. *Cancers (Basel)*. 10.3390/cancers17040705 40002298 10.3390/cancers17040705 PMC11852742

[18] Braxton, A. M., Kiemen, A. L., Grahn, M. P., Forjaz, A., Parksong, J., Mahesh Babu, J., Lai, J., Zheng, L., Niknafs, N., Jiang, L., et al. (2024). 3D genomic mapping reveals multifocality of human pancreatic precancers. *Nature,**629*(8012), 679–687. 38693266 10.1038/s41586-024-07359-3

[19] de la Fuente, J., Chatterjee, A., Lui, J., Nehra, A. K., Bell, M. G., Lennon, R. J., Kassmeyer, B. A., Graham, R. P., Nagayama, H., Schulte, P. J., et al. (2023). Long-term outcomes and risk of pancreatic cancer in intraductal papillary mucinous neoplasms. *JAMA Network Open,**6*(10), Article e2337799. 37847503 10.1001/jamanetworkopen.2023.37799 PMC10582793

[20] Halbrook, C. J., Lyssiotis, C. A., di Pasca Magliano, M., & Maitra, A. (2023). Pancreatic cancer: Advances and challenges. *Cell,**186*(8), 1729–1754. 37059070 10.1016/j.cell.2023.02.014 PMC10182830

[21] Debernardi, S., Liszka, L., Ntala, C., Steiger, K., Esposito, I., Carlotti, E., Baker, A. M., McDonald, S., Graham, T., Dmitrovic, B., et al. (2024). Molecular characteristics of early-onset pancreatic ductal adenocarcinoma. *Molecular Oncology,**18*(3), 677–690. 38145461 10.1002/1878-0261.13576 PMC10920080

[22] Innamorati, G., Valenti, M. T., Giacomello, L., Dalle Carbonare, L., & Bassi, C. (2016). GNAS mutations: Drivers or co-pilots? Yet, promising diagnostic biomarkers. *Trends in Cancer,**2*(6), 282–285. 28741526 10.1016/j.trecan.2016.05.005

[23] Hu, C. M., Tien, S. C., Hsieh, P. K., Jeng, Y. M., Chang, M. C., Chang, Y. T., Chen, Y. J., Chen, Y. J., Lee, E. Y. P., & Lee, W. H. (2019). High glucose triggers nucleotide imbalance through O-GlcNAcylation of key enzymes and induces KRAS mutation in pancreatic cells. *Cell Metabolism,**29*(6), 1334-1349 e1310. 30853214 10.1016/j.cmet.2019.02.005

[24] Suspene, R., Raymond, K. A., Guardado-Calvo, P., Dairou, J., Bonhomme, F., Bonenfant, C., Guyetant, S., Lecomte, T., Pages, J. C., & Vartanian, J. P. (2025). Disruption of deoxyribonucleotide triphosphate biosynthesis leads to RAS proto-oncogene activation and perturbation of mitochondrial metabolism. *The Journal of Biological Chemistry,**301*(2), Article 108117. 39722416 10.1016/j.jbc.2024.108117 PMC11791277

[25] Carpenter, E. S., Elhossiny, A. M., Kadiyala, P., Li, J., McGue, J., Griffith, B. D., Zhang, Y., Edwards, J., Nelson, S., Lima, F., et al. (2023). Analysis of donor pancreata defines the transcriptomic signature and microenvironment of early neoplastic lesions. *Cancer Discovery,**13*(6), 1324–1345. 37021392 10.1158/2159-8290.CD-23-0013 PMC10236159

[26] Makohon-Moore, A. P., Matsukuma, K., Zhang, M., Reiter, J. G., Gerold, J. M., Jiao, Y., Sikkema, L., Attiyeh, M. A., Yachida, S., Sandone, C., et al. (2018). Precancerous neoplastic cells can move through the pancreatic ductal system. *Nature,**561*(7722), 201–205. 30177826 10.1038/s41586-018-0481-8 PMC6342205

[27] Allenson, K., Castillo, J., San Lucas, F. A., Scelo, G., Kim, D. U., Bernard, V., Davis, G., Kumar, T., Katz, M., Overman, M. J., et al. (2017). High prevalence of mutant KRAS in circulating exosome-derived DNA from early-stage pancreatic cancer patients. *Annals of Oncology,**28*(4), 741–747. 28104621 10.1093/annonc/mdx004 PMC5834026

[28] Brancato, L., Osok, D., den Van Bossche, L., Cutsem, E. V., Bates, S. E., den Van Bossche, J., & Bogers, J. (2025). CA19-9 as a dynamic biomarker for continuous monitoring of therapeutic efficacy in pancreatic adenocarcinoma. *Cancers (Basel)*. 10.3390/cancers17243902 41463153 10.3390/cancers17243902 PMC12730893

[29] Clawson, G. A., Matters, G. L., Xin, P., McGovern, C., Wafula, E., dePamphilis, C., Meckley, M., Wong, J., Stewart, L., D’Jamoos, C., et al. (2017). Stealth dissemination" of macrophage-tumor cell fusions cultured from blood of patients with pancreatic ductal adenocarcinoma. *PLoS ONE,**12*(9), Article e0184451. 28957348 10.1371/journal.pone.0184451 PMC5619717

[30] Pei, G., Min, J., Rajapakshe, K. I., Branchi, V., Liu, Y., Selvanesan, B. C., Thege, F., Sadeghian, D., Zhang, D., Cho, K. S., et al. (2025). Spatial mapping of transcriptomic plasticity in metastatic pancreatic cancer. *Nature*. 10.1038/s41586-025-08927-x 40269162 10.1038/s41586-025-08927-x PMC13242257

[31] Werba, G., Weissinger, D., Kawaler, E. A., Zhao, E., Kalfakakou, D., Dhara, S., Wang, L., Lim, H. B., Oh, G., Jing, X., et al. (2023). Single-cell RNA sequencing reveals the effects of chemotherapy on human pancreatic adenocarcinoma and its tumor microenvironment. *Nature Communications,**14*(1), Article 797. 36781852 10.1038/s41467-023-36296-4 PMC9925748

[32] Law, H. C., Lagundzin, D., Clement, E. J., Qiao, F., Wagner, Z. S., Krieger, K. L., Costanzo-Garvey, D., Caffrey, T. C., Grem, J. L., DiMaio, D. J., et al. (2020). The proteomic landscape of pancreatic ductal adenocarcinoma liver metastases identifies molecular subtypes and associations with clinical response. *Clinical Cancer Research,**26*(5), 1065–1076. 31848187 10.1158/1078-0432.CCR-19-1496 PMC7056493

[33] Alver, T. N., Bergholtz, H., Holm, M. B., Dorg, L. T., Skrede, M. L., Kure, E. H., & Verbeke, C. S. (2025). Spatial transcriptomics reveals cancer and stromal cell heterogeneity between center and invasive front of pancreatic cancer. *Modern Pathology,**38*(6), Article 100726. 39889965 10.1016/j.modpat.2025.100726

[34] Huang, L., Guo, Z., Wang, F., & Fu, L. (2021). KRAS mutation: From undruggable to druggable in cancer. *Signal Transduction and Targeted Therapy,**6*(1), Article 386. 34776511 10.1038/s41392-021-00780-4 PMC8591115

[35] Rhim, A. D., Mirek, E. T., Aiello, N. M., Maitra, A., Bailey, J. M., McAllister, F., Reichert, M., Beatty, G. L., Rustgi, A. K., Vonderheide, R. H., et al. (2012). EMT and dissemination precede pancreatic tumor formation. *Cell,**148*(1–2), 349–361. 22265420 10.1016/j.cell.2011.11.025 PMC3266542

[36] Masugi, Y. (2022). The desmoplastic stroma of pancreatic cancer: Multilayered levels of heterogeneity, clinical significance, and therapeutic opportunities. *Cancers (Basel)*. 10.3390/cancers14133293 35805064 10.3390/cancers14133293 PMC9265767

[37] Makareeva, E., Han, S., Vera, J. C., Sackett, D. L., Holmbeck, K., Phillips, C. L., Visse, R., Nagase, H., & Leikin, S. (2010). Carcinomas contain a matrix metalloproteinase-resistant isoform of type I collagen exerting selective support to invasion. *Cancer Research,**70*(11), 4366–4374. 20460529 10.1158/0008-5472.CAN-09-4057 PMC2880213

[38] Panciera, T., Citron, A., Di Biagio, D., Battilana, G., Gandin, A., Giulitti, S., Forcato, M., Bicciato, S., Panzetta, V., Fusco, S., et al. (2020). Reprogramming normal cells into tumour precursors requires ECM stiffness and oncogene-mediated changes of cell mechanical properties. *Nature Materials,**19*(7), 797–806. 32066931 10.1038/s41563-020-0615-x PMC7316573

[39] Salu, P., & Reindl, K. M. (2024). Advancements in preclinical models of pancreatic cancer. *Pancreas,**53*(2), e205–e220. 38206758 10.1097/MPA.0000000000002277 PMC10842038

[40] Aiello, N. M., Rhim, A. D., & Stanger, B. Z. (2016). Orthotopic injection of pancreatic cancer cells. *Cold Spring Harbor Protocols,**2016*(1), Article pdb.prot078360. 26729902 10.1101/pdb.prot078360

[41] Yamaguchi, J., Kokuryo, T., Yokoyama, Y., Ebata, T., Ochiai, Y., & Nagino, M. (2021). Premalignant pancreatic cells seed stealth metastasis in distant organs in mice. *Oncogene,**40*(12), 2273–2284. 33649537 10.1038/s41388-021-01706-8

[42] Rodriguez-Tirado, C., & Sosa, M. S. (2024). How much do we know about the metastatic process? *Clinical & Experimental Metastasis,**41*(4), 275–299. 38520475 10.1007/s10585-023-10248-0 PMC11374507

[43] Perelli, L., Zhang, L., Mangiameli, S., Giannese, F., Mahadevan, K. K., Peng, F., Citron, F., Khan, H., Le, C., Gurreri, E., et al. (2025). Evolutionary fingerprints of epithelial-to-mesenchymal transition. *Nature,**640*(8060), 1083–1092. 40044861 10.1038/s41586-025-08671-2 PMC13537484

[44] Nordgard, O., Lapin, M., Tjensvoll, K., Oltedal, S., Edland, K. H., Neverdahl, N. B., Fostenes, D., Garresori, H., Glenjen, N., Smaaland, R., et al. (2022). Prognostic value of disseminated tumor cells in unresectable pancreatic ductal adenocarcinoma: A prospective observational study. *BMC Cancer,**22*(1), Article 609. 35659265 10.1186/s12885-022-09714-x PMC9166481

[45] Yang, S., Seo, J., Choi, J., Kim, S. H., Kuk, Y., Park, K. C., Kang, M., Byun, S., & Joo, J. Y. (2025). Towards understanding cancer dormancy over strategic hitching up mechanisms to technologies. *Molecular Cancer,**24*(1), Article 47. 39953555 10.1186/s12943-025-02250-9 PMC11829473

[46] Wu, J. S., Jiang, J., Chen, B. J., Wang, K., Tang, Y. L., & Liang, X. H. (2021). Plasticity of cancer cell invasion: Patterns and mechanisms. *Translational Oncology,**14*(1), Article 100899. 33080522 10.1016/j.tranon.2020.100899 PMC7573380

[47] Zanotelli, M. R., Zhang, J., & Reinhart-King, C. A. (2021). Mechanoresponsive metabolism in cancer cell migration and metastasis. *Cell Metabolism,**33*(7), 1307–1321. 33915111 10.1016/j.cmet.2021.04.002 PMC9015673

[48] Zhang, Y., Li, W., Niu, J., Fan, Z., Li, X., & Zhang, H. (2025). Reprogramming of glucose metabolism in pancreatic cancer: Mechanisms, implications, and therapeutic perspectives. *Frontiers in Immunology,**16*, Article 1586959. 40630951 10.3389/fimmu.2025.1586959 PMC12234474

[49] Li, Z., Wang, Q., Huang, X., Yang, M., Zhou, S., Li, Z., Fang, Z., Tang, Y., Chen, Q., Hou, H., et al. (2023). Lactate in the tumor microenvironment: A rising star for targeted tumor therapy. *Frontiers in Nutrition,**10*, Article 1113739. 36875841 10.3389/fnut.2023.1113739 PMC9978120

[50] Imodoye, S. O., & Adedokun, K. A. (2023). EMT-induced immune evasion: Connecting the dots from mechanisms to therapy. *Clinical and Experimental Medicine,**23*(8), 4265–4287. 37966552 10.1007/s10238-023-01229-4

[51] Gu, X. Y., Yang, J. L., Lai, R., Zhou, Z. J., Tang, D., Hu, L., & Zhao, L. J. (2025). Impact of lactate on immune cell function in the tumor microenvironment: Mechanisms and therapeutic perspectives. *Frontiers in Immunology,**16*, Article 1563303. 40207222 10.3389/fimmu.2025.1563303 PMC11979165

[52] Robertson, F. P., Cameron, A., Spiers, H. V. M., Joseph, N., Taylor, E., Ratnayake, B., Jamieson, N. B., & Pandanaboyana, S. (2024). Evidence for molecular subtyping in pancreatic ductal adenocarcinoma: A systematic review. *HPB : the official journal of the International Hepato Pancreato Biliary Association,**26*(5), 609–617. 38401998 10.1016/j.hpb.2024.02.001

[53] Song, Q., Yang, Y., Jiang, D., Qin, Z., Xu, C., Wang, H., Huang, J., Chen, L., Luo, R., Zhang, X., et al. (2022). Proteomic analysis reveals key differences between squamous cell carcinomas and adenocarcinomas across multiple tissues. *Nature Communications,**13*(1), Article 4167. 35851595 10.1038/s41467-022-31719-0 PMC9293992

[54] de Back, T. R., van Hooff, S. R., Sommeijer, D. W., & Vermeulen, L. (2024). Transcriptomic subtyping of gastrointestinal malignancies. *Trends in Cancer,**10*(9), 842–856. 39019673 10.1016/j.trecan.2024.06.007

[55] Lee, J. J., & Yeh, J. J. (2024). Updates in molecular profiling of pancreatic ductal adenocarcinoma. *Surgical Clinics of North America,**104*(5), 939–950. 39237169 10.1016/j.suc.2024.04.001 PMC11377860

[56] Flowers, B. M., Xu, H., Mulligan, A. S., Hanson, K. J., Seoane, J. A., Vogel, H., Curtis, C., Wood, L. D., & Attardi, L. D. (2021). Cell of origin influences pancreatic cancer subtype. *Cancer Discovery,**11*(3), 660–677. 34009137 10.1158/2159-8290.CD-20-0633 PMC8134763

[57] Iovanna, J., Fraunhoffer, N., Urrutia, R., & Dusetti, N. (2025). Understanding the heterogeneity of pancreatic ductal adenocarcinoma. *Translational Oncology,**60*, Article 102479. 40694876 10.1016/j.tranon.2025.102479 PMC12304713

[58] Di Chiaro, P., Nacci, L., Arco, F., Brandini, S., Polletti, S., Palamidessi, A., Donati, B., Soriani, C., Gualdrini, F., Frige, G., et al. (2024). Mapping functional to morphological variation reveals the basis of regional extracellular matrix subversion and nerve invasion in pancreatic cancer. *Cancer Cell,**42*(4), 662-681 e610. 38518775 10.1016/j.ccell.2024.02.017

[59] Park, J. K., Jeong, H. O., Kim, H., Choi, J. H., Lee, E. M., Kim, S., Jang, J., Choi, D. W., Lee, S. H., Kim, K. M., et al. (2024). Single-cell transcriptome analysis reveals subtype-specific clonal evolution and microenvironmental changes in liver metastasis of pancreatic adenocarcinoma and their clinical implications. *Molecular Cancer,**23*(1), Article 87. 38702773 10.1186/s12943-024-02003-0 PMC11067162

[60] Aiello, N. M., Maddipati, R., Norgard, R. J., Balli, D., Li, J., Yuan, S., Yamazoe, T., Black, T., Sahmoud, A., Furth, E. E., et al. (2018). EMT subtype influences epithelial plasticity and mode of cell migration. *Developmental Cell,**45*(6), 681-695 e684. 29920274 10.1016/j.devcel.2018.05.027 PMC6014628

[61] Pickett, M. R., Kamra, M., Castilla-Casadiego, D. A., Noori, S., Matsui, W., Rosales, A. M., Zoldan, J., & Parekh, S. H. (2026). Collagen nanofiber alignment attenuates leader-follower energetic and metabolic differences during collective migration in pancreatic cancer. *Acta Biomaterialia,**209*, 183–193. 41271079 10.1016/j.actbio.2025.11.031

[62] Maddipati, R. (2025). Metastatic heterogeneity in pancreatic cancer: Mechanisms and opportunities for targeted intervention. *Journal of Clinical Investigation*. 10.1172/JCI191943 40662361 10.1172/JCI191943 PMC12259258

[63] Jimenez-Sanchez, A., Persad, S., Hayashi, A., Umeda, S., Sharma, R., Xie, Y., Mehta, A., Park, W., Masilionis, I., Chu, T. et al. (2025). Transcriptomic plasticity is a hallmark of metastatic pancreatic cancer. *Cancer Research*. 10.1158/0008-5472.CAN-25-1117 10.1158/0008-5472.CAN-25-1117 PMC13044532 41379552

[64] Bozoky, B., Fernandez Moro, C., Strell, C., Geyer, N., Heuchel, R. L., Lohr, J. M., Ernberg, I., Szekely, L., Gerling, M., & Bozoky, B. (2021). Stabilization of the classical phenotype upon integration of pancreatic cancer cells into the duodenal epithelium. *Neoplasia,**23*(12), 1300–1306. 34798385 10.1016/j.neo.2021.11.007 PMC8605302

[65] Pantazopoulou, V., Kubota, C. S., Ogawa, S., Gulay, K. C. M., Lin, X., Song, H., Weitz, J. R., Tiriac, H., Lowy, A. M., & Engle, D. D. (2025). Experimental models of pancreas cancer: What has been the impact for precision medicine? *Journal of Clinical Investigation*. 10.1172/JCI191945 40829173 10.1172/JCI191945 PMC12352895

[66] Xu, J., Pham, M. D., Corbo, V., Ponz-Sarvise, M., Oni, T., Ohlund, D., & Hwang, C. I. (2025). Advancing pancreatic cancer research and therapeutics: The transformative role of organoid technology. *Experimental & Molecular Medicine,**57*(1), 50–58. 39814914 10.1038/s12276-024-01378-w PMC11799150

[67] Pitter, K. L., Grbovic-Huezo, O., Joost, S., Singhal, A., Blum, M., Wu, K., Holm, M., Ferrena, A., Bhutkar, A., Hudson, A., et al. (2022). Systematic comparison of pancreatic ductal adenocarcinoma models identifies a conserved highly plastic basal cell state. *Cancer Research,**82*(19), 3549–3560. 35952360 10.1158/0008-5472.CAN-22-1742 PMC9532381

[68] Tamagawa, H., Fujii, M., Togasaki, K., Seino, T., Kawasaki, S., Takano, A., Toshimitsu, K., Takahashi, S., Ohta, Y., Matano, M., et al. (2024). Wnt-deficient and hypoxic environment orchestrates squamous reprogramming of human pancreatic ductal adenocarcinoma. *Nature Cell Biology*. 10.1038/s41556-024-01498-5 39232216 10.1038/s41556-024-01498-5

[69] Matsumoto, K., Fujimori, N., Ichihara, K., Takeno, A., Murakami, M., Ohno, A., Kakehashi, S., Teramatsu, K., Ueda, K., Nakata, K., et al. (2024). Patient-derived organoids of pancreatic ductal adenocarcinoma for subtype determination and clinical outcome prediction. *Journal of Gastroenterology,**59*(7), 629–640. 38684511 10.1007/s00535-024-02103-0 PMC11217054

[70] Kumano, K., Nakahashi, H., Louphrasitthiphol, P., Kuroda, Y., Miyazaki, Y., Shimomura, O., Hashimoto, S., Akashi, Y., Mathis, B. J., Kim, J., et al. (2024). Hypoxia at 3D organoid establishment selects essential subclones within heterogenous pancreatic cancer. *Frontiers in Cell and Developmental Biology,**12*, Article 1327772. 38374892 10.3389/fcell.2024.1327772 PMC10875002

[71] Landon-Brace, N., Latour, S., Innes, B. T., Nurse, M., Cadavid, J. L., Co, I. L., Tan, C. M., Nowlan, F., Drissler, S., Notta, F., et al. (2025). Analysis of an engineered organoid model of pancreatic cancer identifies hypoxia as a contributing factor in determining transcriptional subtypes. *Science and Reports,**15*(1), 23610. 10.1038/s41598-025-98344-x PMC12223171 40604231

[72] Gout, J., Ekizce, M., Roger, E., & Kleger, A. (2025). Pancreatic organoids as cancer avatars for true personalized medicine. *Advanced Drug Delivery Reviews,**224*, Article 115642. 40582468 10.1016/j.addr.2025.115642

[73] Messal, H. A., Alt, S., Ferreira, R. M. M., Gribben, C., Wang, V. M., Cotoi, C. G., Salbreux, G., & Behrens, A. (2019). Tissue curvature and apicobasal mechanical tension imbalance instruct cancer morphogenesis. *Nature,**566*(7742), 126–130. 30700911 10.1038/s41586-019-0891-2 PMC7025886

[74] Hayashi, A., Fan, J., Chen, R., Ho, Y. J., Makohon-Moore, A. P., Lecomte, N., Zhong, Y., Hong, J., Huang, J., Sakamoto, H., et al. (2020). A unifying paradigm for transcriptional heterogeneity and squamous features in pancreatic ductal adenocarcinoma. *Nature Cancer,**1*(1), 59–74. 35118421 10.1038/s43018-019-0010-1 PMC8809486

[75] Dang, T. T., Esparza, M. A., Maine, E. A., Westcott, J. M., & Pearson, G. W. (2015). DeltaNp63alpha promotes breast cancer cell motility through the selective activation of components of the epithelial-to-mesenchymal transition program. *Cancer Research,**75*(18), 3925–3935. 26292362 10.1158/0008-5472.CAN-14-3363 PMC4573836

[76] Karras, P., Black, J. R. M., McGranahan, N., & Marine, J. C. (2024). Decoding the interplay between genetic and non-genetic drivers of metastasis. *Nature,**629*(8012), 543–554. 38750233 10.1038/s41586-024-07302-6

[77] Knox, J. J., Jang, G. H., Grant, R. C., Zhang, A., Ma, L., Elimova, E., Jang, R., Moore, M., Biagi, J., Tehfe, M., et al. (2025). Whole genome and transcriptome profiling in advanced pancreatic cancer patients on the COMPASS trial. *Nature Communications,**16*(1), Article 5919. 40593593 10.1038/s41467-025-60808-z PMC12219293

[78] Drizyte-Miller, K., Talabi, T., Somasundaram, A., Cox, A. D., & Der, C. J. (2025). KRAS: The Achilles’ heel of pancreas cancer biology. *Journal of Clinical Investigation*. 10.1172/JCI191939 40829181 10.1172/JCI191939 PMC12352898

[79] Wang, L., Wei, D., Li, S., & Jiang, S. (2025). Advances in biomarkers of resistance to KRAS mutation-targeted inhibitors. *Discovery Oncology,**16*(1), Article 1834. 10.1007/s12672-025-03569-x PMC12508421 41060473

[80] Dilly, J., Hoffman, M. T., Abbassi, L., Li, Z., Paradiso, F., Parent, B. D., Hennessey, C. J., Jordan, A. C., Morgado, M., Dasgupta, S., et al. (2024). Mechanisms of resistance to oncogenic KRAS inhibition in pancreatic cancer. *Cancer Discovery,**14*(11), 2135–2161. 38975874 10.1158/2159-8290.CD-24-0177 PMC11528210

[81] Singhal, A., Styers, H. C., Rub, J., Li, Z., Torborg, S. R., Kim, J. Y., Grbovic-Huezo, O., Feng, H., Tarcan, Z. C., Sahin Ozkan, H., et al. (2024). A classical epithelial state drives acute resistance to KRAS inhibition in pancreatic cancer. *Cancer Discovery,**14*(11), 2122–2134. 38975873 10.1158/2159-8290.CD-24-0740 PMC11624508

[82] Wang, S., Zheng, Y., Yang, F., Zhu, L., Zhu, X. Q., Wang, Z. F., Wu, X. L., Zhou, C. H., Yan, J. Y., Hu, B. Y., et al. (2021). The molecular biology of pancreatic adenocarcinoma: Translational challenges and clinical perspectives. *Signal Transduction and Targeted Therapy,**6*(1), Article 249. 34219130 10.1038/s41392-021-00659-4 PMC8255319

